# Multiparametric MRI radiomics in prostate cancer for predicting Ki-67 expression and Gleason score: a multicenter retrospective study

**DOI:** 10.1007/s12672-023-00752-w

**Published:** 2023-07-20

**Authors:** Chuan Zhou, Yun-Feng Zhang, Sheng Guo, Dong Wang, Hao-Xuan Lv, Xiao-Ni Qiao, Rong Wang, De-Hui Chang, Li-Ming Zhao, Feng-Hai Zhou

**Affiliations:** 1 The First Clinical Medical College of Lanzhou University, Lanzhou, 73000 China; 2 The First Clinical Medical College of Gansu University of Chinese Medicine, Lanzhou, 730000 China; 3 Department of Urology, Gansu Provincial Hospital, Lanzhou, 730000 China; 4 Department of Nuclear Medicine, Gansu Provincial Hospital, Lanzhou, 730000 China; 5 Department of Information Management, Gansu Provincial Hospital, Lanzhou, 730000 China; 6 Department of Urology, The 940 Hospital of Joint Logistics Support Force of Chinese PLA, Lanzhou, 730000 China; 7 Department of Urology, Second People’s Hospital of Gansu Province, Lanzhou, 730000 China

**Keywords:** Prostate cancer, Magnetic resonance imaging (MRI), Ki-67, Gleason score, Radiomics, Machine learning

## Abstract

**Purpose:**

Prostate cancer (PCa) with high Ki-67 expression and high Gleason Scores (GS) tends to have aggressive clinicopathological characteristics and a dismal prognosis. In order to predict the Ki-67 expression status and the GS in PCa, we sought to construct and verify MRI-based radiomics signatures.

**Methods and materials:**

We collected T2-weighted imaging (T2WI), diffusion-weighted imaging (DWI), and apparent diffusion coefficient (ADC) images from 170 PCa patients at three institutions and extracted 321 original radiomic features from each image modality. We used support vector machine (SVM) and least absolute shrinkage and selection operator (LASSO) logistic regression to select the most informative radiomic features and built predictive models using up sampling and feature selection techniques. Using receiver operating characteristic (ROC) analysis, the discriminating power of this feature was determined. Subsequent decision curve analysis (DCA) assessed the clinical utility of the radiomic features. The Kaplan–Meier (KM) test revealed that the radiomics-predicted Ki-67 expression status and GS were prognostic factors for PCa survival.

**Result:**

The hypothesized radiomics signature, which included 15 and 9 selected radiomics features, respectively, was significantly correlated with pathological Ki-67 and GS outcomes in both the training and validation datasets. Areas under the curve (AUC) for the developed model were 0.813 (95% CI 0.681,0.930) and 0.793 (95% CI 0.621, 0.929) for the training and validation datasets, respectively, demonstrating discrimination and calibration performance. The model's clinical usefulness was verified using DCA. In both the training and validation sets, high Ki-67 expression and high GS predicted by radiomics using SVM models were substantially linked with poor overall survival (OS).

**Conclusions:**

Both Ki-67 expression status and high GS correlate with PCa patient survival outcomes; therefore, the ability of the SVM classifier-based model to estimate Ki-67 expression status and the Lasso classifier-based model to assess high GS may enhance clinical decision-making**.**

**Supplementary Information:**

The online version contains supplementary material available at 10.1007/s12672-023-00752-w.

## Introduction

With a global incidence rate of 13.5% and a mortality rate of 6.7%, PCa ranks as the fifth leading cause of death in males worldwide [1, 2]. Imaging techniques have gone a long way in recent years, and this has major implications on PCa diagnosis, detection, screening, staging of the illness, response to therapy and individualized prediction of oncological outcomes [3–5]. Radiology has revolutionized the cancer diagnostic process. Imaging diagnostics provide the noninvasive and cost-effective method for scalable detection, localization, and monitoring of cancer. Imaging modalities outside radiography, such as skin pictures and colonoscopy films, are also used for screening and diagnosis [6].In the past, qualitative evaluation of imaging modalities was prioritized, but as artificial intelligence (AI) techniques for analyzing radiological characteristics have advanced, the emphasis of radiomics has shifted to quantitative data measurement from these images [7]. The therapeutic relevance of "radiomics" is also being explored and has the propensity to be extended to clinical applications by merging image characteristics and phenotypes with gene and protein signatures [8, 9].

The nuclear DNA binding protein Ki-67 is a standard for tumor staging present in all vertebrates [10]. Several nuclear cell cycle study investigations have shown that Ki-67 is only present in actively dividing (G1, S, and G2) cells and not in resting cells (G0). Therefore, Ki-67 might function as a cell proliferation marker in several malignancies [11]. Immunohistochemistry (IHC) evaluation of paraffin-embedded tissue sections for Ki-67 labeling index and percent positive frequency are predictive of clinical outcomes for individual patients. Generally speaking, a poor prognosis might be expected in clinical situations when the Ki-67 score is high [12, 13]. To aid in the prognostic assessment of individuals with PCa, prostate biopsy samples are graded according to the Gleason system [14]. A higher Gleason score (GS) indicates a more aggressive form of PCa and a worse prognosis for the patient. Pathological ratings vary from 2 to 10, with higher values suggesting more dangers and higher mortality [15]. The GSs score reflects the extent of infiltration and invasion of PCa and is a crucial reference for clinical urologists, becoming part of the TNM or Whitmore-Jewett PCa staging system to provide a prognosis. According to Mayo’s research, using GS, perineural invasion, and Ki-67 expression together is the most accurate way to predict long-term outcomes from PCa [16]. Given its cheap cost, fast evaluation, and high predictive value, Matthew.T et al. suggested that the addition of Ki-67 expression to perineural invasion and GS at biopsy should be regarded as a benchmark against which all novel biomarkers are evaluated before their introduction into clinical practice [16]. However, Ki-67 expression and GS of PCa can only be acquired by transrectal ultrasound-guided prostate biopsy (TRUS-Bx) or radical prostatectomy highlighting the need for a non-invasive and more reliable means of prognosis.

Magnetic resonance imaging (MRI) is a valuable technique for PCa radiomics research because it can identify clinical PCas, conduct lesion-targeted biopsies with high sensitivity and specificity, and provide high-quality pictures [17]. Radiomics is a method that extracts quantitative features from medical images to reveal tumor characteristics and heterogeneity. MRI-based radiomics is a helpful tool for PCa diagnosis because of its ability to predict PCa features such as GS, Prostate Imaging Reporting and Data System version 2.1 (PIRADS v2), risk class, and automated prostate segmentation inside a completely automated quality control system [18, 19]. Compared to imaging characteristics derived by subjective judgment, radiomics is more objective and may extract high-dimensional imaging features that are not visible to the human and may be connected with intratumor heterogeneity [20]. T2-WI may reveal the architecture of the prostate, as well as the lesion's location, size, shape, and extent, as well as the existence of perineural invasion or seminal vesicle invasion [21]. In general, PCa has a low signal on T2-WI, although cancer foci in the transitional zone may be difficult to differentiate from benign hyperplastic lesions. DWI may reflect the diffusion of water molecules in the tissue, and prostate cancer often exhibits a strong signal on DWI due to its high cell density and poor diffusivity of water molecules [21]. DWI may increase the detection rate and localization precision of prostate cancer, particularly in the transitional and central zones [22]. ADC is a quantitative parameter of DWI that reflects the diffusion coefficient of water molecules in tissues. ADC is inversely correlated to DWI, that is, the lower the ADC, the higher the DWI signal. ADC can help eliminate the effect of T2 signal enhancement (T2 shine-through) on DWI and improve the assessment of the malignancy degree (Gleason score) of prostate cancer [23]. It is suggested that the T2-WI, DWI, and ADC-based radiomics features can indicate the biological behavior and heterogeneity on the onset of tumor and may facilitate the application of Ki-67 expression and GS prediction in PCa.

Radiomics has been applied to predict Ki-67 expression in bladder cancer using MRI-based features [24], such as T2-weighted (T2-WI) and dynamic contrast-enhancement images. In addition, Ahmad C. et al. predicted GS of PCa patients using radiomics which included T2-WI and apparent diffusion coefficient (ADC, computed from diffusion-weighted imaging) scans of 99 PCa patients [25]. This study showed random forest (RF) classifier model was used to predict GS groups and identify the most important signature among the 41 radiomic features. It concluded that the radiomic features could be used as a non-invasive biomarker to predict the GS of PCa patients [25]. Yanjie Zhao et al. investigated whether radiological and radiomic variables from magnetic resonance imaging might predict a Ki-67 proliferative index in meningioma patients using a machine learning (ML) model. The prediction model might help treat meningioma patients by predicting their Ki-67 score. AUCs of 0.837 and 0.700 showed good prediction models [26].

To better utilize the predictive power of radiomics, we developed a radiomics prediction model through a multicenter collaboration. we adopted the radiomics to extract 318 features from T2-WI, DWI, and ADC images and used SVM and LASSO algorithms to construct radiomics signatures to preoperatively predict the Ki-67 expression and GS status and investigate their prognostic value in PCa.

## Materials and methods

With the approval of the local institution's ethical committee, retrospective clinical-pathologic data on patients with PCa was acquired from electronic medical systems in the three centers (Center A, Gansu Provincial Hospital; Center B, The 940 Hospital of Joint Logistics Support Force of Chinese PLA; Center C, Second People's Hospital of Gansu Province). This retrospective study was also approved by the Ethics Committee of the Gansu Provincial Geriatrics Association (2022-61) and the requirement for informed consent was waived. Furthermore, our research program was patterned around a local institution’s AI model.

### Participants

In our multicenter retrospective cohort analysis, 574 PCa patients with a main pathology diagnosis, between January 1 2017, and January 1, 2022, were included based on the inclusion and exclusion criteria. The inclusion criteria included the following: (a) PCa was diagnosed with histopathology and IHC; (b) All MRI scans were performed within 30 days of PCa diagnosis to exclude the confounding effects of medications on the measurements; (c) Completed prognosis information; (d) No missing information of Ki-67 and GS. The exclusion criteria included the following: (a) Inability to identify the location of the tumor in MRI (Figure S1); (b) Chemotherapy and radiotherapy were performed before pelvic MRI; (c) Lesions for which the borders were difficult to determine; (d) Incomplete clinical information.

**Fig. 1:**
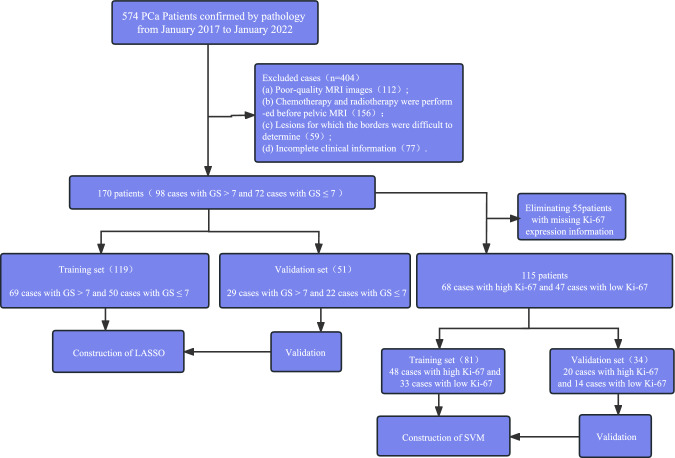
Patient selection flow chart. *SVM* support vector machine, *LASSO* Least absolute shrinkage and selection operator

We gathered the data from a total of 574 PCa-diagnosed patients based on the aforementioned inclusion criteria. From January 1 2017 to January 1, 2022, data from a total of 399 Center A patients were inducted into the training cohort. Furthermore, from August 2018 to May 2020, data from 97 patients with PCa from Center B and data from 78 patients from Center C were included in a testing cohort. Figure 1 depicted the flowchart for patient recruiting (Fig. 1).

### Prostate tumor segmentation

**Fig. 2:**
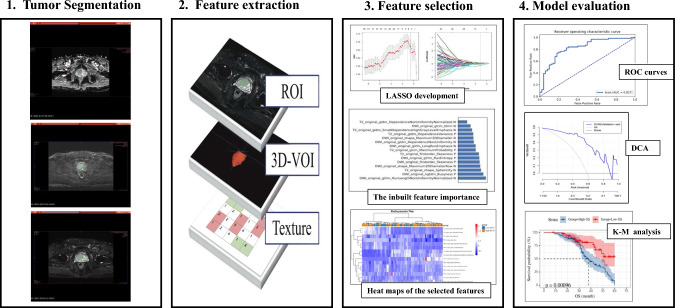
Workflow for the radiomics process. **a** MR segmentation of the region of interest (ROIs) on T2-weighted images (T2WI), DWI, ADC. **b** Extraction of radiomics features within the defined volumes of interest (VOIs) to quality global features, texture features, and nontexture features. **c** Radiomics feature construction using the least absolute shrinkage and selection operator (LASSO) regression. d Predictive performance assessment with respect to discrimination, calibration, and decision curve analysis. **d** Predictive performance assessment with respect to discrimination, decision curve analysis, K-M

One radiologist (with over 5-year experience in pelvic MRI reading) and one urologist (with over 30-year experience in prostate MRI reading) segmented the region of interest (ROI) (Figure S2). If there were any objections to the findings, they would be debated until an agreement was established. Two radiologists were unaware of the prognostic information, Ki-67 expression, and GS throughout this procedure (Fig. 2). For each PCa patient, the boundaries of the tumor were drawn on each slice on the T2WI images, DWI images, and ADC images using ITK-SNAP software (version 4.0.0; http://itk-snap.org). When a patient had numerous tumors, the largest lesion was segmented for feature extraction. Volumes of interest (VOI) was created by stacking each patient's ROIs. To prevent data heterogeneity, Digital Imaging and Communications in Medicine (DICOM) which is the standard file format for the management of medical imaging information and related data were normalized and resampled to the same resolution (1 mm × 1 mm × 1 mm) before radiomics feature extraction. Patients were randomly divided into training and test sets at a ratio of 7:3.

### Feature extraction

After segmentation was complete, the PyRadiomics program (http://www.radiomics.io/pyradiomics.html) was used for lesion feature extraction. For feature extraction, we employed FeAture Explorer (FAE), a visualization program (Figure S3). It uses Python, NumPy, pandas, and scikit-learning modules. FAE can extract image characteristics, preprocess the feature matrix, create alternative models automatically, and assess them with standard clinical statistics [27]. First-order, form, and texture features make up the raw feature classes. Due to a single MRI sequence containing 107 features, mp-MRI produced 321 features in total for this study. In total 321 radiomics features were recovered from the axial T2WI, DWI, and ADC images. All patients' radiomics characteristics were normalized using the Z-score [(x –μ)/σ]. x is the radiomics feature value, is the mean of the feature values, and is the standard deviation of the feature values and was computed using the training set.

### Feature selection

Z-score was used to standardize the dataset. Normalization is the process of subtracting the mean value and dividing the standard deviation for each characteristic. In the course of developing the histology classification model for this research, we up-sampled the positive and negative samples to reduce the imbalance in the classification training set data. To decrease the dimensions of the row space of the feature matrix, we used a Pearson Correlation Coefficient (PCC) for every pair of features [26]. If the PCC was more than 0.9, we randomly eliminated one of them. Multiple iterative calculations enable classifiers to determine the optimal feature combination by evaluating the importance of features and determining the optimal feature combination. Therefore, recursive feature elimination (RFE) based on LASSO and SVM was used to find the optimal feature combination based on accuracy step by step. To determine the hyper-parameter (e.g., the number of features) of model, we applied cross-validation with fivefold on the training data set. The hyper-parameters were set according to the model performance on the validation data set.

### Evaluation of Ki-67 and Gleason score

After surgical resection or TRUS-Bx, IHC was performed on PCa samples for assessment of the Ki-67 and GS within 7 days. Ki-67 expression was detected using Anti-Ki-67 Rabbit pAb (GB111499) according to the manufacturer's procedure. Two independent pathologists who were blind to prognostic and clinical information assessed the immunoreactivity for Ki-67 based on the proportion of Ki-67-positive cells among 1000 cells randomly picked from each section. Similarly, GS was based on the morphology of cancer cells in Hematoxylin and eosin stain (HE) prostate puncture biopsies or resection specimens. After analyzing the tissue samples, the pathologist then assigned a grade to the observed patterns of the tumor specimen:(a) Primary grade—assigned to the dominant pattern of the tumor (has to be greater than 50% of the total pattern seen). (b)Secondary grade assigned to the next-most frequent pattern (has to be less than 50%, but at least 5% of the pattern of total cancer observed). The pathologist then summed the pattern number of the primary and secondary grades to obtain the final GS. The GS in this study were all total scores. Based on studies relating to the prognosis of PCa [1, 27–31], the following criteria were applied: high Ki-67 expression group (> 10% cells stained) and low Ki-67 (≤ 10% cells stained) expression group; high GS group (> 7) and low GS group (≤ 7).

### Model evaluation

Calculated the radiomics score(rad-score) for each sample in the classification model [28, 29]. Applied the sigmoidal function to convert the rad-scores into the probability of high Ki-67 expression and high GS scores P (The value range was 0 ~ 1). The receiver operating characteristic (ROC) curves and the area under the curve measured the diagnostic efficacy of the models (AUC). Based on the truncation value that maximizes the Youden index value to calculate the diagnostic compliance, sensitivity, and specificity of the model. Moreover, the study of decision curves (DCA) demonstrated the clinical net gains offered by the prediction model.

### Statistical analysis

SPSS 23.0 (SPSS, Armonk, NY, USA) and R statistical software (version 3.6.1 R, https://www.r-project.org/) were used for statistical analysis. The R modules utilized in this investigation were indicated in the Supplemental information (Tables 1, 2). The clinical features of the training and validation sets were compared using the appropriate Student's t-test, Chi-square test, or Mann–Whitney U test. The Kaplan–Meier and log-rank tests were conducted across two groups, which were determined by the IHC-based Ki-67 expression status and the radiomics-predicted Ki-67 expression status, respectively. All tests were 2-tailed, and a P < 0.05 was considered as statistically significant.

**Table 1:** Clinical characteristics and Ki-67 in the training cohort and testing cohort

Characteristic	Train (n = 81)		*P* value	Test (n = 34)		*P* value
	Ki-67 ≤ 10 (n = 33)	Ki-67 > 10 (n = 48)		Ki-67 ≤ 10 (n = 14)	Ki-67 > 10 (n = 20)	
Age (year)						
< 65	9	6	0.092^a^	4	3	0.335^a^
≥ 65	24	42		10	17	
Bone metastasis						
Yes	10	19	0.392^a^	3	9	0.157^a^
No	23	29		11	11	
BMI (kg/m^2^)						
18.5 ≤ BMI < 25	19	30	0.575^b^	8	9	0.647^b^
25 ≤ BMI < 30	12	17		5	11	
30 ≤ BMI < 35	2	1		1	0	
PSA (ng/ml)						
0–4	5	2	0.011^b^	1	1	0.635^b^
4–10	7	4		2	2	
> 10	21	42		11	17	
T						
T1	7	7	0.124^b^	2	1	0.738^b^
T2	20	25		7	13	
T3	5	11		4	2	
T4	1	5		1	4	
N						
N0	29	28	0.004^a^	12	11	0.06^a^
N1	4	20		2	9	

*BMI* body mass index, *PSA* Prostate specific antigen

^a^Statistical analysis performed using chi-square test

^b^Statistical analysis performed using Mann–Whitney U test

**Table 2:** Clinical characteristics and GS in the training cohort and testing cohort

Characteristic	Train (n = 119)		*P* value	Test (n = 51)		*P* value
	GS ≤ 7 (n = 50)	GS > 7 (n = 69)		GS ≤ 7 (n = 22)	GS > 7 (n = 29)	
Age(year)						
< 65	15	12	0.105^a^	4	5	0.931^a^
≥ 65	35	57		18	24	
Bone metastasis						
Yes	11	37	< 0.001a	2	22	< 0.001^a^
No	39	32		20	7	
BMI(kg/m^2^)						
18.5 ≤ BMI < 25	28	49	0.095^b^	13	22	0.179^b^
25 ≤ BMI < 30	21	19		8	7	
30 ≤ BMI < 35	1	1		1	0	
PSA(ng/ml)						
0–4	7	1	< 0.001^b^	1	1	0.002^b^
4–10	15	2		8	0	
> 10	28	66		13	28	
T						
T1	19	2	< 0.001^b^	8	0	< 0.001^b^
T2	27	43		9	7	
T3	2	15		4	20	
T4	2	9		1	2	
N						
N0	43	36	< 0.001^a^	18	15	0.026^a^
N1	7	33		4	14	

*BMI* body mass index, *PSA* Prostate specific antigen

^a^Statistical analysis performed using chi-square test

^b^Statistical analysis performed using Mann–Whitney U test

## Result

### Clinical characteristics

This multicenter retrospective analysis comprised 574 participants with a mean age of 72 ± 7 years and a range of 45–90 years. Following the study's inclusion and exclusion criteria, we eliminated 404 participants and included information from 170 patients. There were 98 cases with GS > 7 and 72 cases with GS ≤ 7 included.

After eliminating 54 samples with missing Ki-67 expression information from the enrolled data, a total of 115 samples were used in the development of the Ki-67 prediction model, comprising 68 instances with KI-67 > 10% and 47 cases with Ki-67 10%. Age, bone metastasis, BMI, and T-stage statistics were not significantly different between the two groups (Tables 1, 2). Ki-67 and GS are independent risk factors affecting the prognosis of prostate cancer patients (Tables S1, S2).

### Feature selection and construction of radiomics signatures

**Fig. 3:**
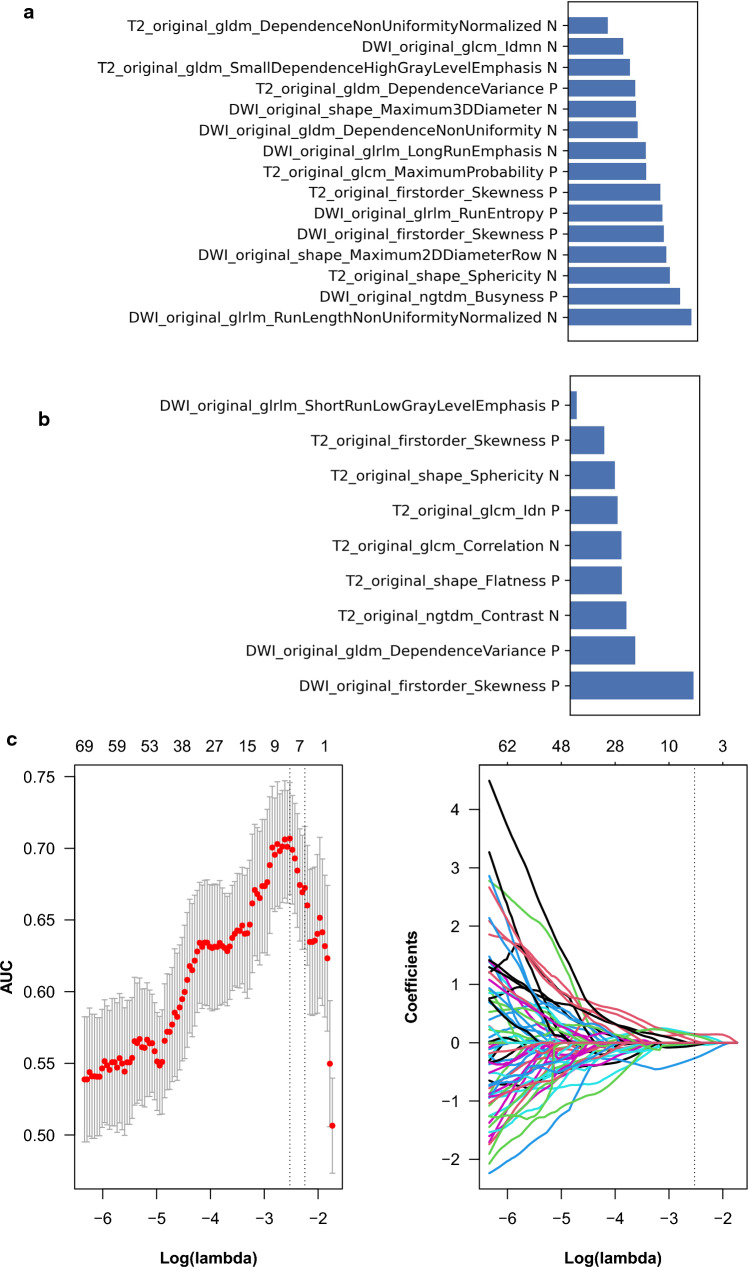
The inbuilt feature importance in the SVM (**a**) and LASSO (**b**) the development of the LASSO in training set (**c**)

**Fig. 4:**
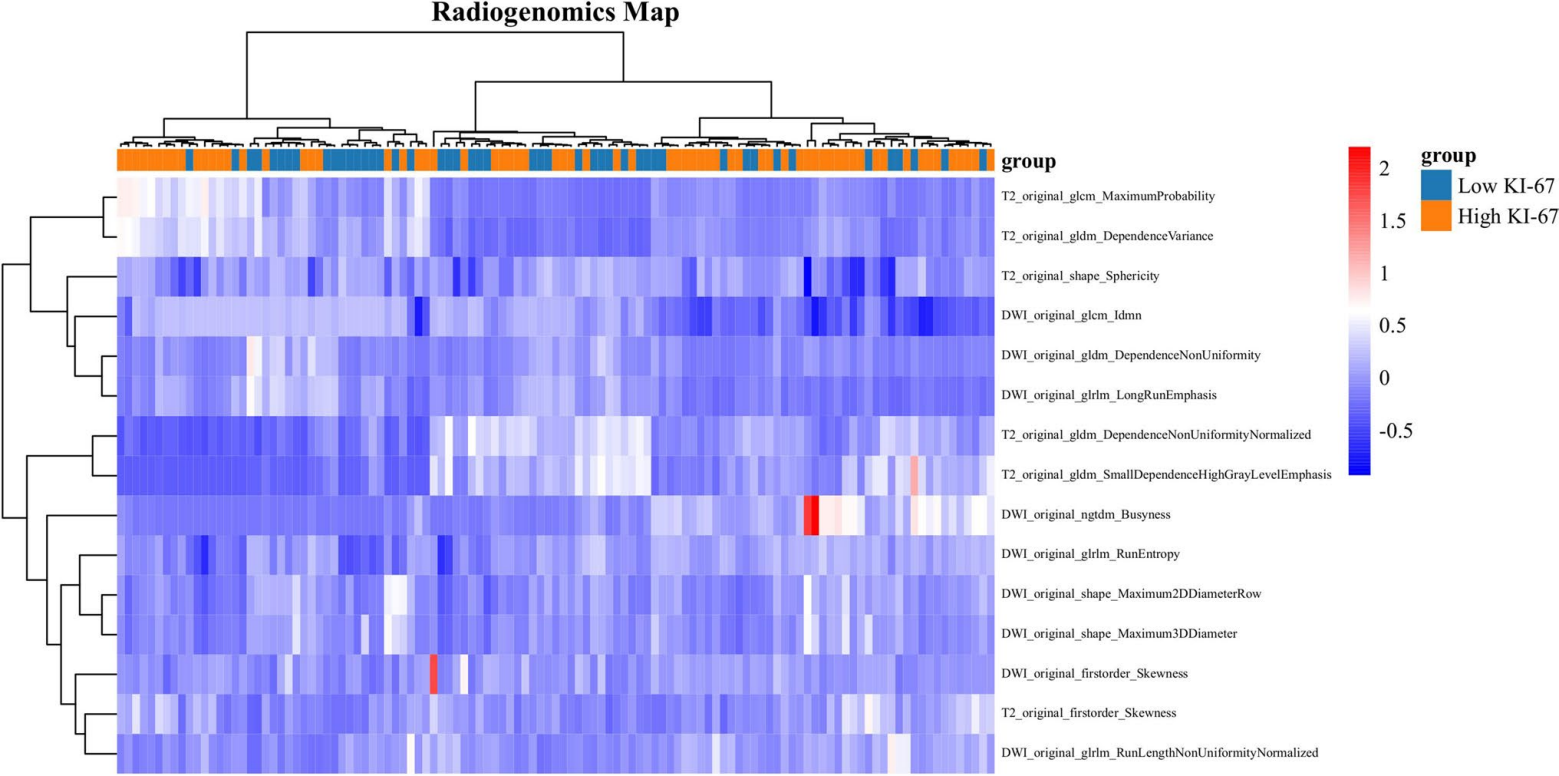
Heat maps of the selected features. The color of the maps represented the value of the selected features. The color of positive cases or high Ki-67 expression cases was generally darker. This proved the ability of the features themselves to distinguish the biological characteristics of patients

The results of feature extraction and construction of the prediction model are as follows:

- We performed feature extraction after feature outlining of the incorporated MRI images. Types of features extracted included the shape, gray level co-occurrence matrix (GLCM), histogram (intensity-based or first-order), gray level size zone matrix (GLSZM), and level run length matrix (GLRLM). 321 features were extracted from each PCa ROI.
- A total of 321 features were retrieved from the three levels of T2-WI, DWI, and ADC, and after deleting individual faulty fields, 318 features were used in the subsequent analysis**.** In the process of building the histological classification model in this study, to eliminate the imbalance of the classification training set data (48/33 ratio of high expression to low expression cases), we balanced the positive and negative samples by up-sampling.
- The Z-score was used to normalize the feature matrix, and due to the high spatial dimensionality of the extracted features, PCC was used to dimensionally reduce the data. The feature vectors of the transformed feature matrix were independent of each other, and the RFE algorithm was used to perform feature selection and rank the features before building the model. The top 15 features were chosen as the best feature subset for the Ki-67 classification model, while the top 9 features were chosen for the GS classification model (Fig. 3). Feature-chosen heat maps. The maps' hues indicated the relative importance of the highlighted elements. Positive cases, or those with elevated Ki-67 expression, were often a deeper hue. This exhibited the features' capacity to differentiate patients based on their biological properties (Fig. 4).
- SVM and LASSO were chosen as the images used by the classifier to construct the classification models for Ki-67 and GS, respectively. For imaging radiomics modeling and testing, the corresponding samples were separated into training and test sets (81/34, 119/51) in a 7:3 ratio. To define the model parameters, a 5-folder cross-validation was performed to test the model's classification performance, and the optimal model parameters were ultimately chosen. The final Ki-67 and GS classification models for PCa were developed by linearly combining the best features with their respective coefficients.

### Performance of prediction models

**Fig. 5:**
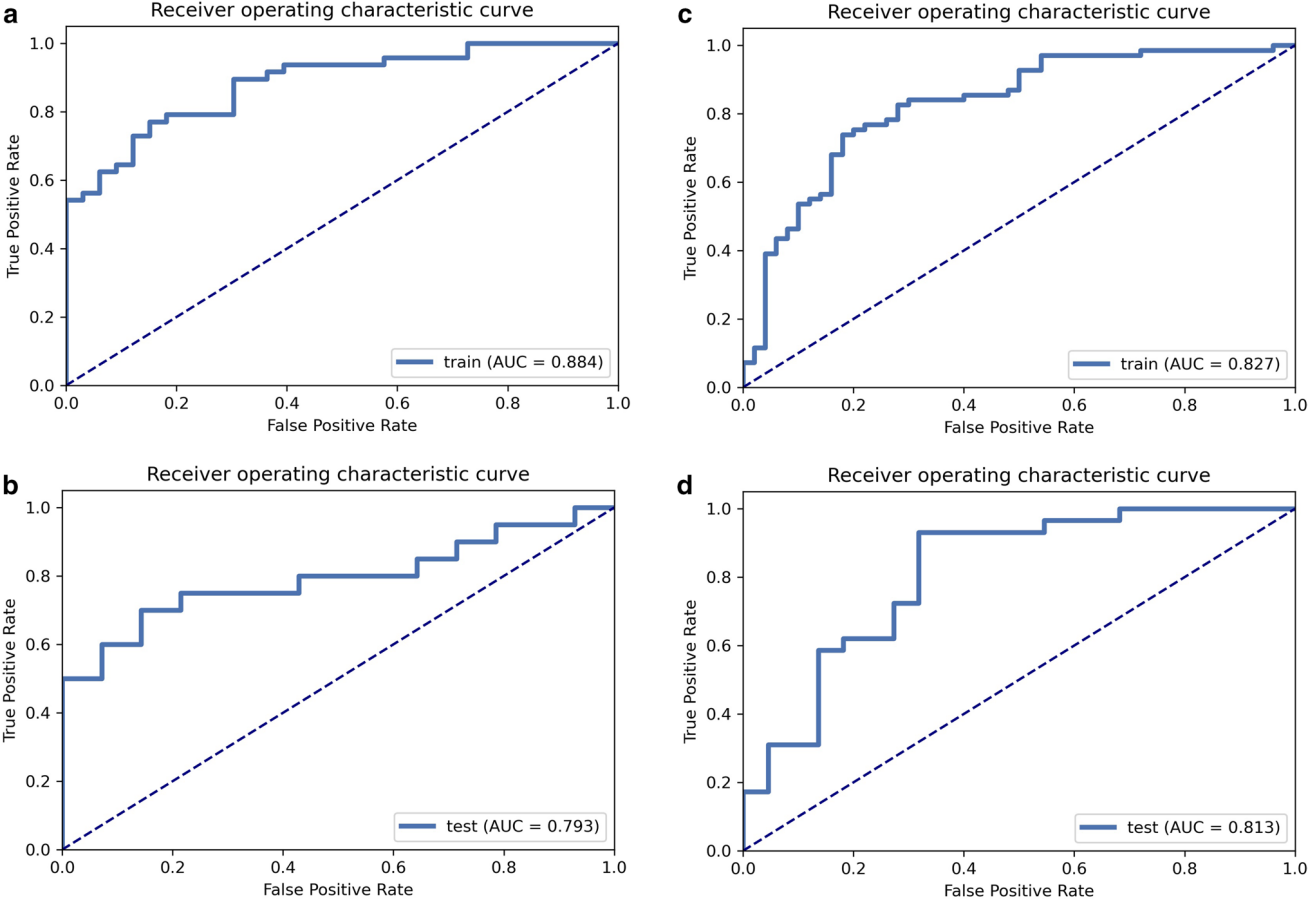
The performance of models. The ROC curves of SVM in training sets (**a**) and validation sets (**b**).The ROC curves of Lasso in training sets (**c**) and validation sets (**d**). *ROC* receiver operating curve, *AUC* area under the ROC curve

**Fig. 6:**
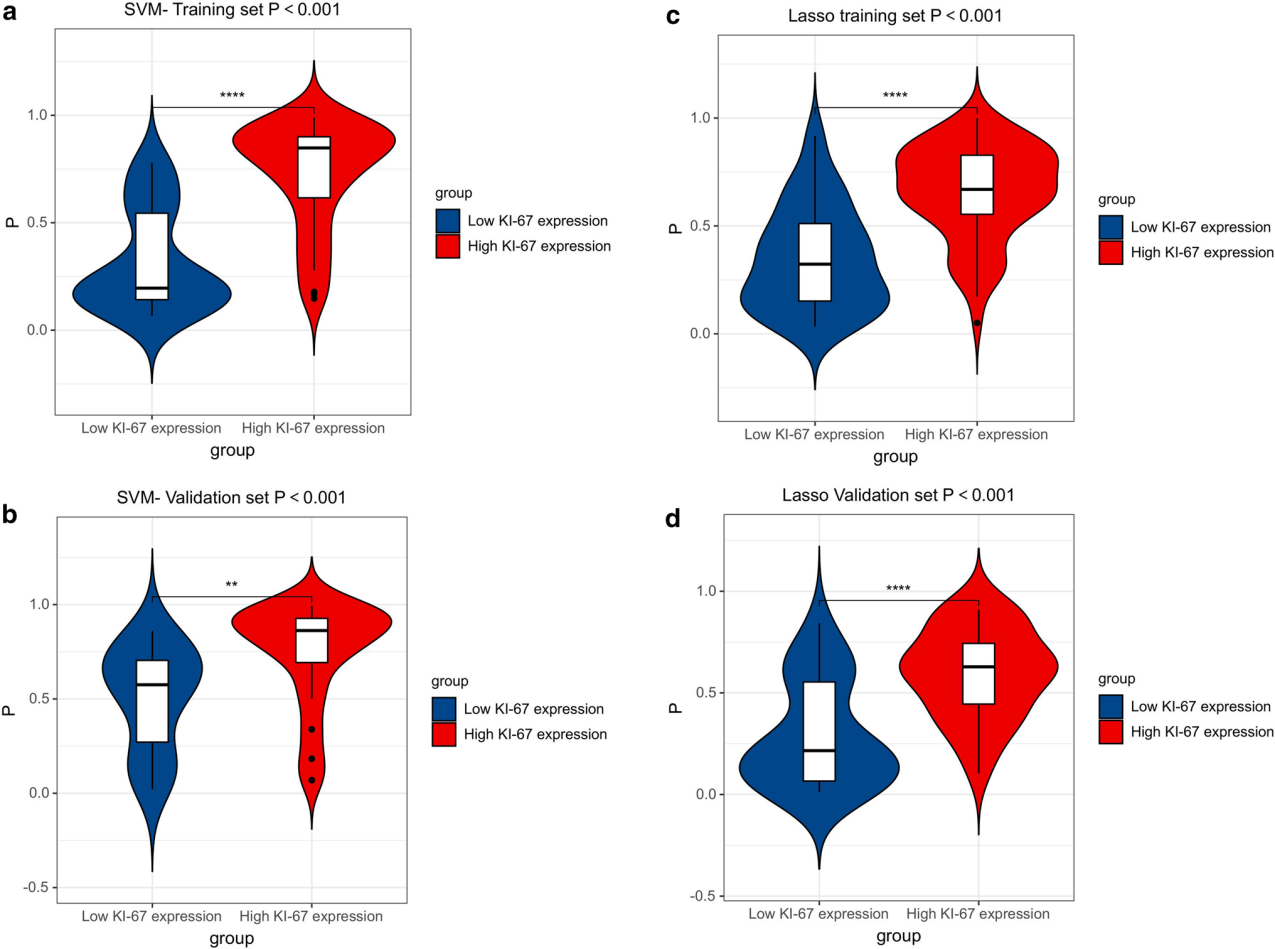
Violin plot. Violin plot of probability of SVM model based on training set (**a**) and validation set (**b**); Violin plot of probabilities of a LASSO model based on training set (**c**) and validation set (**d**)

**Fig. 7:**
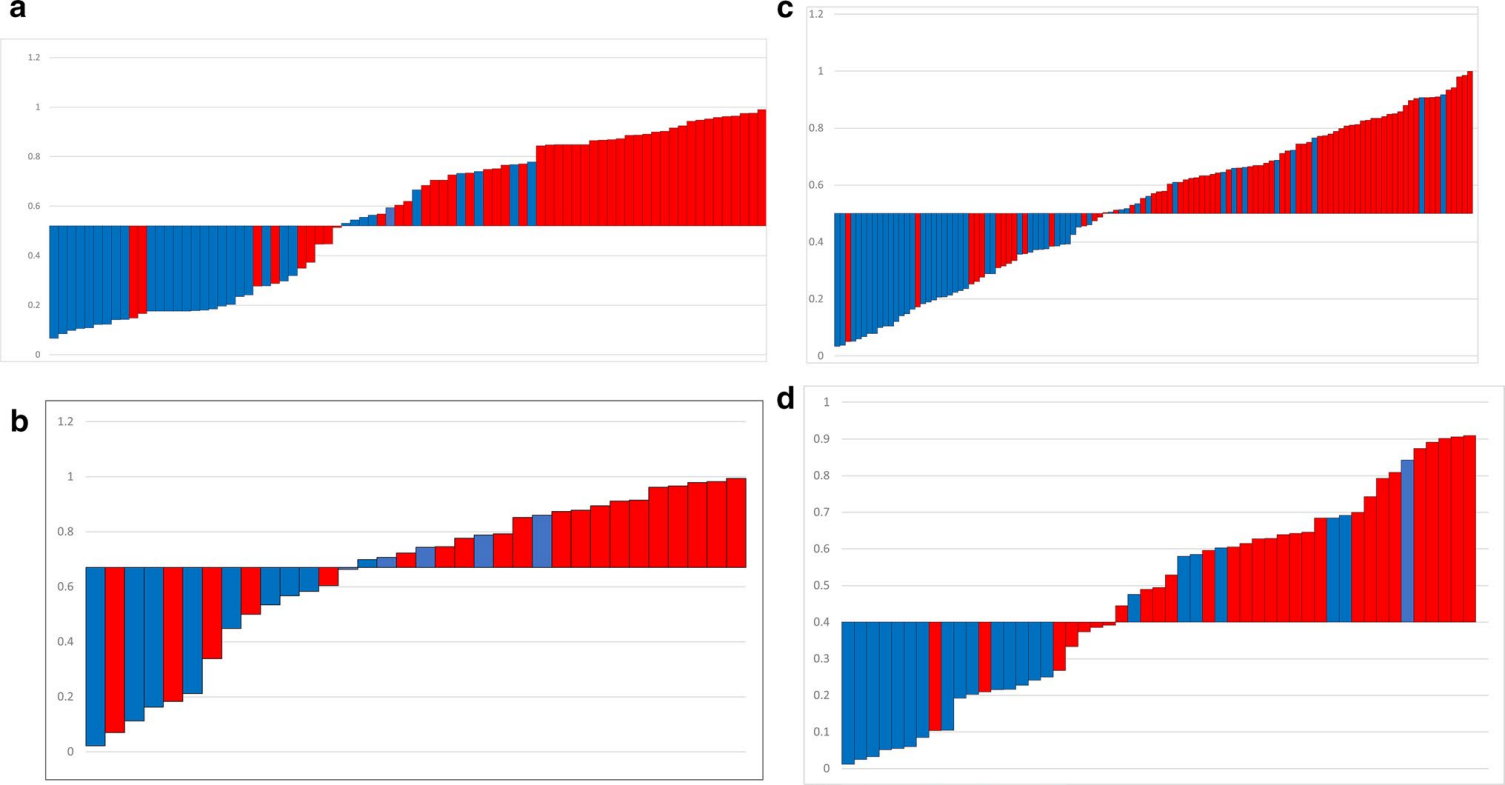
The predicted value for each sample in the models. The predicted value for each sample in the training set (**a**) and validation set (**b**) (SVM); The predicted value for each sample in the training set (**c**) and validation set (**d**) (Lasso)

**Fig. 8:**
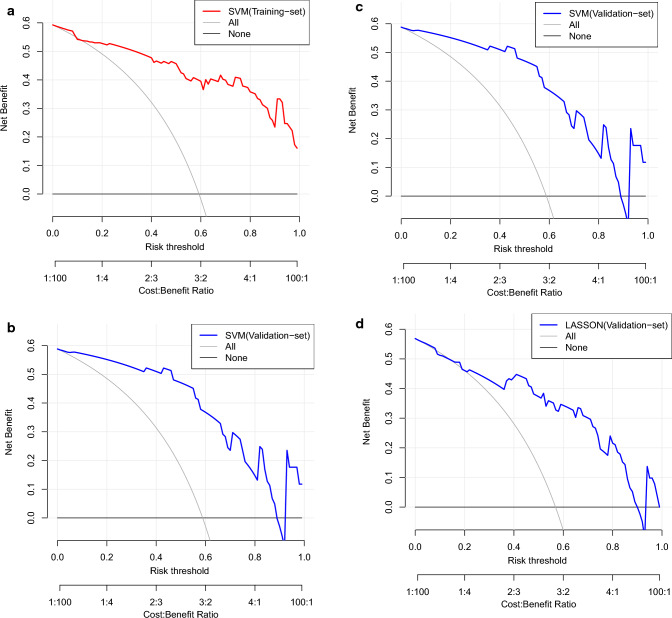
Decision Curve Analyses. DCA for radiomics signature in SVM-training (**a**) and validation sets (**b**); DCA for radiomics signature in Lasso-training (**b**) and validation sets (**d**); DCA: decision curve analyses

The optimal classification model for the Ki-67 employs SVM as the classifier, and its AUCs on the training and validation sets were 0.884 and 0.793, respectively (Fig. 5a, b). The GS optimal classification model employs LASSO as the classifier, and its AUCs on the training and validation sets were 0.827 and 0.813 respectively (Fig. 5c, d) (Tables 3, 4). In models, the estimated probabilities for each sample were considerably greater for the high-expression and high-risk groups in the training and validation sets than for the low-expression and low-risk groups (Figs. 6, 7). DCA demonstrated that the imaging histology characteristics had a positive net clinical effect in both datasets (Fig. 8).

**Table 3:** Clinical statistics (GS) in the diagnosis

Statistics	Value
Accuracy	0.824
AUC	0.814
AUC 95% Cis	[0.681–0.930]
NPV	0.882
PPV	0.794
Sensitivity	0.931
Specificity	0.682

*AUC* area under the ROC curve, *NPV* Negative Predictive Value, *PPV* Positive Predictive Value, *CIs* confidence intervals

**Table 4:** Clinical statistics (Ki-67) in the diagnosis

Statistics	Value
Accuracy	0.765
AUC	0.793
AUC 95% CIs	[0.621–0.929]
NPV	0.667
PPV	0.875
Sensitivity	0.700
Specificity	0.857

*AUC* area under the ROC curve, *NPV* Negative Predictive Value, *PPV* Positive Predictive Value, *CIs* confidence intervals

### Prognosis

#### Relationship between Ki-67 expression status and OS

**Fig. 9:**
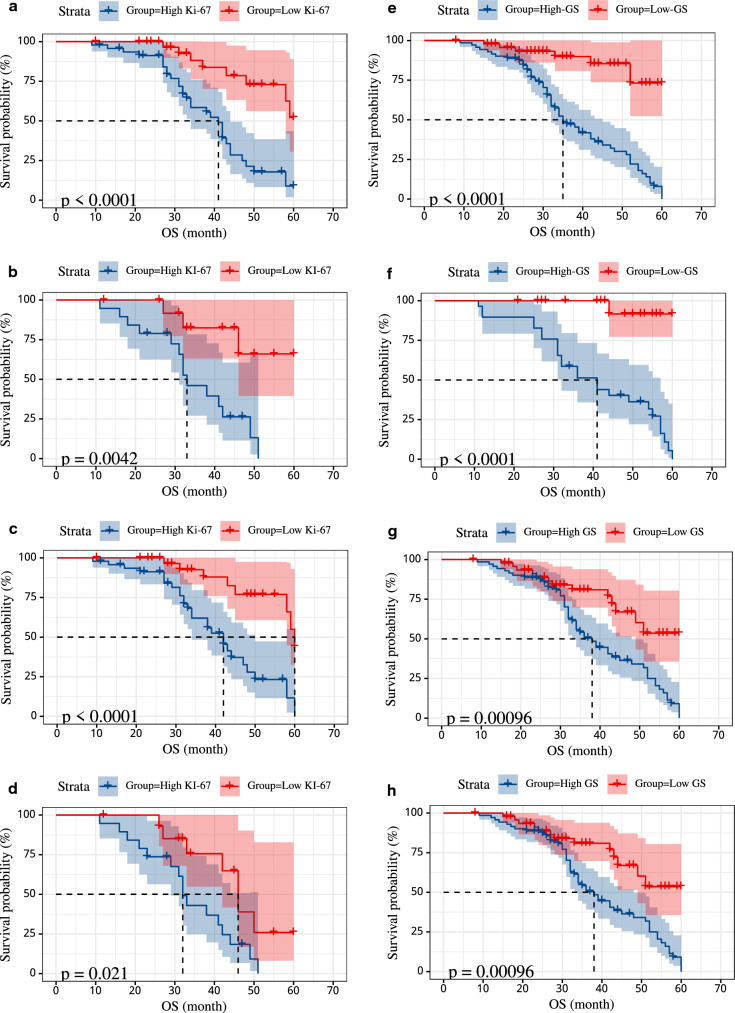
Kaplan–Meier OS Curves. Prognostic value of the IHC-based Ki-67 expression status and radiomics-predicted Ki-67 expression status based on the SVM model. Kaplan–Meier OS curves for patients grouped by IHC-based Ki-67 expression status in training sets (**a**) and validation sets (**b**). Kaplan–Meier OS curves for patients grouped by radiomics-predicted Ki-67 expression status in training sets (**c**) and validation sets (**d**). Prognostic value of the Pathology-based gleason and radiomics-predicted gleason based on the SVM model. Kaplan–Meier OS curves for patients grouped by Pathology-based gleason in training sets (**e**) and validation sets (**f**). Kaplan–Meier OS curves for patients grouped by radiomics-predicted gleason in training sets (**g**) and validation sets (**h**). *IHC* immunohistochemistry, *OS* disease-free survival

- In the Ki-67 classification model study, a total of 55 patients (47%) experienced tumor progression-related events. OS was significantly lower in IHC-Ki-67-high-expression patients than in IHC-Ki-67-low expression patients within both the training and validation groups (Fig. 9a, b; P < 0.05)
- The SVM model's relationship between OS and Ki-67 expression predicted by Radiomics showed that PCa patients with Rad-high-Ki-67-expression had significantly lower OS than patients with Rad-low-Ki-67-expression in both the training and validation groups (Fig. 9c, d; P < 0.05).

#### Relationship between Gleason score and OS

- In the GS classification model study, a total of 86 patients (51%) experienced tumor progression-related events. OS was significantly lower in patients with IHC-GS-high-expression than in patients with IHC-GS-low expression in both the training and validation groups (Fig. 9e, f; P < 0.05)
- The SVM model's relationship between OS and GS predicted by Radiomics showed that PCa patients with Rad-high-GS-expression significantly lower OS than Rad-low-GS-expression in both the training and validation group (Fig. 9 g, h; P < 0.05)

## Discussion

In this multicenter retrospective study, we constructed and validated MRI-based radiomics signatures for the prediction of Ki-67 expression status and GS in PCa. To remove the imbalance of the training data set, we used up-sampling by repeating random cases to make positive/negative samples balance. We applied the normalization on the feature matrix. Without data balancing, the predictive performance of radiomics signatures was inadequate, with obviously low specificity. After data balancing by the up-sampling, the synthesized performance of radiomics signatures was further improved, indicating that data balancing contributes to constructing more powerful prediction models. For the Ki-67 classifier, we settled on a set of 15 features, while for the GS classifier, we narrowed it down to a set of 9. Prediction of Ki-67 expression status and the GS was best for the LASSO and SVM in this investigation, and DCA showed that this model produced good clinical net benefit. Therefore, radiomics based on MRI might aid in predicting the Ki-67 expression and the GS in PCa.

Due to the rising uses of radiomics in oncology and its efficacy in differentiating malignancies, which is difficult with conventional radiologic interpretations, the use of radiomics is interesting [30–32]. Radiomics illuminates the invisible link between diseased outcomes and extracted variables, hence facilitating clinical decision-making [20]. Radiomics postulates that the most important information is included in the spatial distribution of voxel intensities [28]. Indeed, our preliminary results demonstrated that four dominant radiomics categories from T2-WI and DWI of the selected texture 318 features could quantify intratumor heterogeneity, and that these categories were consistent with those from prior studies that reported the relevant features that are predictors for staging PCa aggressiveness [29, 33]. With the use of more potent algorithmic models, the top radiomic characteristics presented in this research may aid in giving more accurate predictions. Moreover, by exploring similarities or differences in tumor heterogeneity genes at the microscopic level, several key factors are provided for the development of improved radiomics models prediction for hallmarks of PCa, proliferation, and aggressiveness.

Computer-aided diagnostics (CAD) is a system that employs ML algorithms to analyze imaging and/or non-imaging medical data and assess the patient’s condition, which can be used to assist physicians in decision-making [34]. RF, SVM, and LASSO algorithms are the CAD tools commonly used to classify resampled instances to analyze medical images from picture databases [35]. In this work, we tested SVM, LDA, RF, and LASSO to find the best model, and finally selected SVM and LASSO for predicting Ki-67 expression and GS in the data based on their higher predictive accuracy. This was also an exploration of the application of CAD. As clinical urologists, we are more interested in the algorithmic maturity of ML or DL, as well as the scope and applicability of clinical applications. The SVM and LASSO models that we have chosen from the results of the existing studies have different application characteristics in the CAD of PCa. SVM has the best accuracy in distinguishing GS in image augmentation [36], the ability to handle high-dimensional data, and good generalization ability [37, 38]. However, it also has drawbacks such as high training and testing costs, sensitivity to parameters, and difficulty in interpretation [39]. On the other hand, the advantage of LASSO is that it can extract features and regularize radiomics data simultaneously to prevent overfitting, and it is suitable for correlated high-dimensional data [40, 41]. However, it may lose some important features and be sensitive to outliers. In contrast, direct comparisons between different ML or DL algorithms are scarce in terms of available research. Most of the above-mentioned studies did not compare their classifiers, so a conclusion about the best algorithm classifier to be used for the study of radiomics in PCa cannot be drawn at this time.

Our predictive models built on machine learning SVM and LR-Lasso classifiers are suitable for processing small sample data with high classification accuracy and are particularly suitable for solving dichotomous classification problems. The models are relatively simple and easy to understand and implement but may require fine-grained feature engineering and tuning of parameters. At the same time, the data requirements are relatively relaxed and can handle problems such as classification, regression, and anomaly detection, provided that the data quality is good. In contrast, deep learning models, such as VGG and Resnet models [42, 43], are generally used for image classification and target detection tasks in large-scale datasets and usually require more computational resources and time for multi-classification problems [44]. At the same time, their requirements for data quality are higher and require normalization and pre-processing, which may otherwise have an impact on the effectiveness of the models. In conclusion, machine learning models and deep learning models each have their own characteristics and are suitable for different scenarios and problems [45]. The correct choice of the appropriate algorithm requires a comprehensive consideration of the actual task and dataset characteristics, including data volume, data nature, classification accuracy requirements, and computational resources, to achieve the best classification results [46].

Our results corresponded with the prognosis of PCa patients has been the final argument. In previous retrospective clinical research, Ki-67 and GS showed a strong association with the prognosis of patients with PCa [10, 47]. Our findings are identical to those of previous research, but we emphasize the non-invasive, repeatable, and user-friendly character of radiomics. In this study, we aimed to investigate the association among PCa patient Ki-67 expression status, the GS, and survival outcomes (5-year OS) predicted by radiomics using the LASSO and SVM. Our model predicted high Ki-67 expression, high GS in PCa patients with considerably reduced Ki-67 expression (Fig. 9c, d), and low GS in patients with OS (Fig. 9g, h), as shown by the data. The foregoing findings may imply that the model we built may directly predict the prognosis of patients using MRI data, therefore providing urologists with more important information for clinical decision-making.

The present study has some limitations, partly due to the exploratory nature of assessing the feasibility of the new application domain. (a) First, this is a retrospective multicenter analysis with a relatively small sample size. A prospective multicenter study with a larger sample size is needed to validate our results for future clinical applications. (b) The complementary role of radiomics signatures in the established predictive model is worth exploring, which will be our future direction to provide a more robust model. (c)In this study, we classified patients with a GS (Gleason score) of 4 + 3 as low risk. However, it is important to note that the prognosis for these patients is significantly worse compared to those with a GS of 3 + 4, placing them in the high-risk group from a clinical perspective. This discrepancy can be attributed mainly to our limited sample size. If we had categorized these patients as high risk, the disparity in the number of positive and negative samples (83/36) would have been too substantial, significantly impacting the reliability of our results. (d) Although the findings are promising and the models performed well, the inherent uncertainty should be considered before applying this information in clinical settings.

## Conclusion

Initial findings indicate that radiomic features derived from MRI imaging are a reliable tool for predicting Ki-67 overexpression and high GS. The radiomic properties performed well when examined for discrimination, calibration, and clinical utility. The proposed noninvasive technique has the potential to enhance clinical decision making for individuals with PCa.

## Supplementary Information

**Additional file 1. **

**Additional file 2. **

**Additional file 3. **

**Additional file 4.**

**Additional file 5.**

## Data Availability

All data supporting the findings of this study are available within the paper and its Supplementary material.

## Abbreviations

ADC: Apparent diffusion coefficient
AUC: Area under the receiver operating curve
DCA: Decision curve analyses
DWI: Diffusion-weighted imaging
GS: Gleason score
HE: Hematoxylin and eosin stain
IHC: Immunohistochemistry
KM: Kaplan–Meier
LASSO: Least absolute shrinkage and selection operator
MRI: Magnetic resonance imaging
OS: Disease-free survival
PCas: Prostate cancers (PCa singular)
PIRADS: Prostate imaging reporting and data system
ROC: Receiver operating curve
SVM: Support vector machine
T2-WI: T2-weighted imaging

## References

[1] Bray F, Ferlay J, Soerjomataram I, Siegel RL, Torre LA, Jemal A (2018). Global cancer statistics 2018: GLOBOCAN estimates of incidence and mortality worldwide for 36 cancers in 185 countries. CA Cancer J Clin.

[2] Sung H, Ferlay J, Siegel RL, Laversanne M, Soerjomataram I, Jemal A (2021). Global cancer statistics 2020: GLOBOCAN estimates of incidence and mortality worldwide for 36 cancers in 185 Countries. CA Cancer J Clin.

[3] Bruno SM, Falagario UG, d'Altilia N, Recchia M, Mancini V, Selvaggio O (2021). PSA density help to identify patients with elevated PSA due to prostate cancer rather than intraprostatic inflammation: a prospective single center study. Front Oncol.

[4] Maggi M, Gentilucci A, Salciccia S, Gatto A, Gentile V, Colarieti A (2019). Psychological impact of different primary treatments for prostate cancer: a critical analysis. Andrologia.

[5] Logozzi M, Angelini DF, Giuliani A, Mizzoni D, Di Raimo R, Maggi M (2019). Increased plasmatic levels of PSA-expressing exosomes distinguish prostate cancer patients from benign prostatic hyperplasia: a prospective study. Cancers (Basel).

[6] Hosny A, Parmar C, Quackenbush J, Schwartz LH, Aerts H (2018). Artificial intelligence in radiology. Nat Rev Cancer.

[7] Smith CP, Czarniecki M, Mehralivand S, Stoyanova R, Choyke PL, Harmon S (2019). Radiomics and radiogenomics of prostate cancer. Abdom Radiol (NY).

[8] Kumar V, Gu Y, Basu S, Berglund A, Eschrich SA, Schabath MB (2012). Radiomics: the process and the challenges. Magn Reson Imaging.

[9] Lambin P, Rios-Velazquez E, Leijenaar R, Carvalho S, van Stiphout RG, Granton P (2012). Radiomics: extracting more information from medical images using advanced feature analysis. Eur J Cancer.

[10] Sobecki M, Mrouj K, Colinge J, Gerbe F, Jay P, Krasinska L (2017). Cell-cycle regulation accounts for variability in Ki-67 expression levels. Cancer Res.

[11] Urruticoechea A, Smith IE, Dowsett M (2005). Proliferation marker Ki-67 in early breast cancer. J Clin Oncol.

[12] Perou CM, Jeffrey SS, van de Rijn M, Rees CA, Eisen MB, Ross DT (1999). Distinctive gene expression patterns in human mammary epithelial cells and breast cancers. Proc Natl Acad Sci U S A.

[13] Mesko S, Kupelian P, Demanes DJ, Huang J, Wang PC, Kamrava M (2013). Quantifying the ki-67 heterogeneity profile in prostate cancer. Prostate Cancer.

[14] Male Genital Pathology. The Internet Pathology Laboratory for Medical Education. The University of Utah, Eccles Health Sciences Library.

[15] Ruela-de-Sousa RR, Hoekstra E, Hoogland AM, Souza Queiroz KC, Peppelenbosch MP, Stubbs AP (2016). Low-molecular-weight protein tyrosine phosphatase predicts prostate cancer outcome by increasing the metastatic potential. Eur Urol.

[16] Tollefson MK, Karnes RJ, Kwon ED, Lohse CM, Rangel LJ, Mynderse LA (2014). Prostate cancer Ki-67 (MIB-1) expression, perineural invasion, and gleason score as biopsy-based predictors of prostate cancer mortality: the Mayo model. Mayo Clin Proc.

[17] Bevilacqua A, Mottola M, Ferroni F, Rossi A, Gavelli G, Barone D (2021). The primacy of high B-Value 3T-DWI radiomics in the prediction of clinically significant prostate cancer. Diagnostics (Basel).

[18] Gugliandolo SG, Pepa M, Isaksson LJ, Marvaso G, Raimondi S, Botta F (2021). MRI-based radiomics signature for localized prostate cancer: a new clinical tool for cancer aggressiveness prediction? Sub-study of prospective phase II trial on ultra-hypofractionated radiotherapy (AIRC IG-13218). Eur Radiol.

[19] Sunoqrot M, Selnæs KM, Sandsmark E, Nketiah GA, Zavala-Romero O, Stoyanova R (2020). A quality control system for automated prostate segmentation on T2-weighted MRI. Diagnostics (Basel).

[20] Aerts HJ, Velazquez ER, Leijenaar RT, Parmar C, Grossmann P, Carvalho S (2014). Decoding tumour phenotype by noninvasive imaging using a quantitative radiomics approach. Nat Commun.

[21] Abdellaoui A, Iyengar S, Freeman S (2011). Imaging in prostate cancer. Future Oncol.

[22] Yağci AB, Ozari N, Aybek Z, Düzcan E (2011). The value of diffusion-weighted MRI for prostate cancer detection and localization. Diagn Interv Radiol.

[23] Thompson J, Lawrentschuk N, Frydenberg M, Thompson L, Stricker P, USANZ (2013). The role of magnetic resonance imaging in the diagnosis and management of prostate cancer. BJU Int.

[24] Zheng Z, Gu Z, Xu F, Maskey N, He Y, Yan Y (2021). Magnetic resonance imaging-based radiomics signature for preoperative prediction of Ki67 expression in bladder cancer. Cancer Imaging.

[25] Chaddad A, Niazi T, Probst S, Bladou F, Anidjar M, Bahoric B (2018). Predicting Gleason score of prostate cancer patients using radiomic analysis. Front Oncol.

[26] Zhao Y, Xu J, Chen B, Cao L, Chen C (2022). Efficient prediction of Ki-67 proliferation index in meningiomas on MRI: from traditional radiological findings to a machine learning approach. Cancers (Basel).

[27] Song Y, Zhang J, Zhang YD, Hou Y, Yan X, Wang Y (2020). FeAture Explorer (FAE): a tool for developing and comparing radiomics models. PLoS One.

[28] Ma S, Xie H, Wang H, Yang J, Han C, Wang X (2020). Preoperative prediction of extracapsular extension: radiomics signature based on magnetic resonance imaging to stage prostate cancer. Mol Imaging Biol.

[29] Ma S, Xie H, Wang H, Han C, Yang J, Lin Z (2019). MRI-based radiomics signature for the preoperative prediction of extracapsular extension of prostate cancer. J Magn Reson Imaging.

[30] Nketiah G, Elschot M, Kim E, Teruel JR, Scheenen TW, Bathen TF (2017). T2-weighted MRI-derived textural features reflect prostate cancer aggressiveness: preliminary results. Eur Radiol.

[31] Xie H, Zhang X, Ma S, Liu Y, Wang X (2019). Preoperative differentiation of uterine sarcoma from leiomyoma: comparison of three models based on different segmentation volumes using radiomics. Mol Imaging Biol.

[32] Huang YQ, Liang CH, He L, Tian J, Liang CS, Chen X (2016). Development and validation of a radiomics nomogram for preoperative prediction of lymph node metastasis in colorectal cancer. J Clin Oncol.

[33] Wang J, Wu CJ, Bao ML, Zhang J, Wang XN, Zhang YD (2017). Machine learning-based analysis of MR radiomics can help to improve the diagnostic performance of PI-RADS v2 in clinically relevant prostate cancer. Eur Radiol.

[34] Chan HP, Hadjiiski LM, Samala RK (2020). Computer-aided diagnosis in the era of deep learning. Med Phys.

[35] Yoo S, Gujrathi I, Haider MA, Khalvati F (2019). Prostate cancer detection using deep convolutional neural networks. Sci Rep.

[36] Makowski MR, Bressem KK, Franz L, Kader A, Niehues SM, Keller S (2021). De Novo radiomics approach using image augmentation and features from T1 mapping to predict Gleason scores in prostate cancer. Invest Radiol.

[37] Zhao W, Xu Y, Yang Z, Sun Y, Li C, Jin L (2019). Development and validation of a radiomics nomogram for identifying invasiveness of pulmonary adenocarcinomas appearing as subcentimeter ground-glass opacity nodules. Eur J Radiol.

[38] Huang Y, Liu Z, He L, Chen X, Pan D, Ma Z (2016). Radiomics signature: a potential biomarker for the prediction of disease-free survival in early-stage (I or II) non-small cell lung cancer. Radiology.

[39] Sushentsev N, Rundo L, Blyuss O, Nazarenko T, Suvorov A, Gnanapragasam VJ (2022). Comparative performance of MRI-derived PRECISE scores and delta-radiomics models for the prediction of prostate cancer progression in patients on active surveillance. Eur Radiol.

[40] Tibshirani R (1997). The lasso method for variable selection in the Cox model. Stat Med.

[41] Ji X, Zhang J, Shi W, He D, Bao J, Wei X (2021). Bi-parametric magnetic resonance imaging based radiomics for the identification of benign and malignant prostate lesions: cross-vendor validation. Phys Eng Sci Med.

[42] Ben Hamida A, Devanne M, Weber J, Truntzer C, Derangère V, Ghiringhelli F (2021). Deep learning for colon cancer histopathological images analysis. Comput Biol Med.

[43] Noorbakhsh J, Farahmand S, Foroughi Pour A, Namburi S, Caruana D, Rimm D (2020). Deep learning-based cross-classifications reveal conserved spatial behaviors within tumor histological images. Nat Commun.

[44] Zhou J, Zhang Y, Chang KT, Lee KE, Wang O, Li J (2020). Diagnosis of benign and malignant breast lesions on DCE-MRI by using radiomics and deep learning with consideration of peritumor tissue. J Magn Reson Imaging.

[45] Tran KA, Kondrashova O, Bradley A, Williams ED, Pearson JV, Waddell N (2021). Deep learning in cancer diagnosis, prognosis and treatment selection. Genome Med.

[46] Skrede OJ, De Raedt S, Kleppe A, Hveem TS, Liestøl K, Maddison J (2020). Deep learning for prediction of colorectal cancer outcome: a discovery and validation study. Lancet.

[47] Zhao L, Yu N, Guo T, Hou Y, Zeng Z, Yang X (2014). Tissue biomarkers for prognosis of prostate cancer: a systematic review and meta-analysis. Cancer Epidemiol Biomarkers Prev.

