# Supplementary figures and images for: Multiparametric MRI radiomics in prostate cancer for predicting Ki-67 expression and Gleason score: a multicenter retrospective study
Source: Discov Oncol. 2023 Jul 20;14:133. doi: 10.1007/s12672-023-00752-w (PMC10361451; doi:10.1007/s12672-023-00752-w)

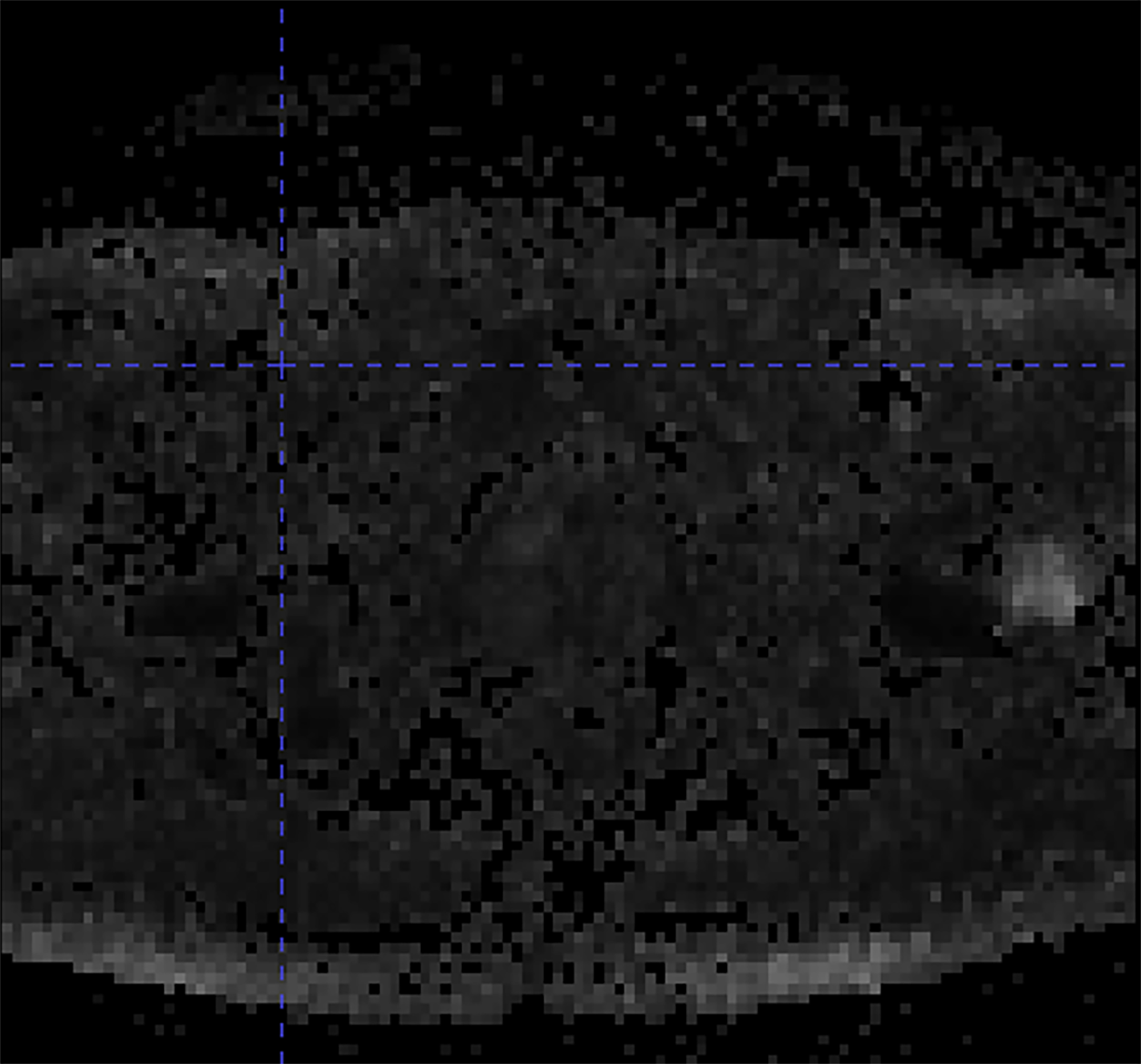

Supplement: Supplementary file 1 — Additional file 1. [file 12672_2023_752_MOESM1_ESM.png]

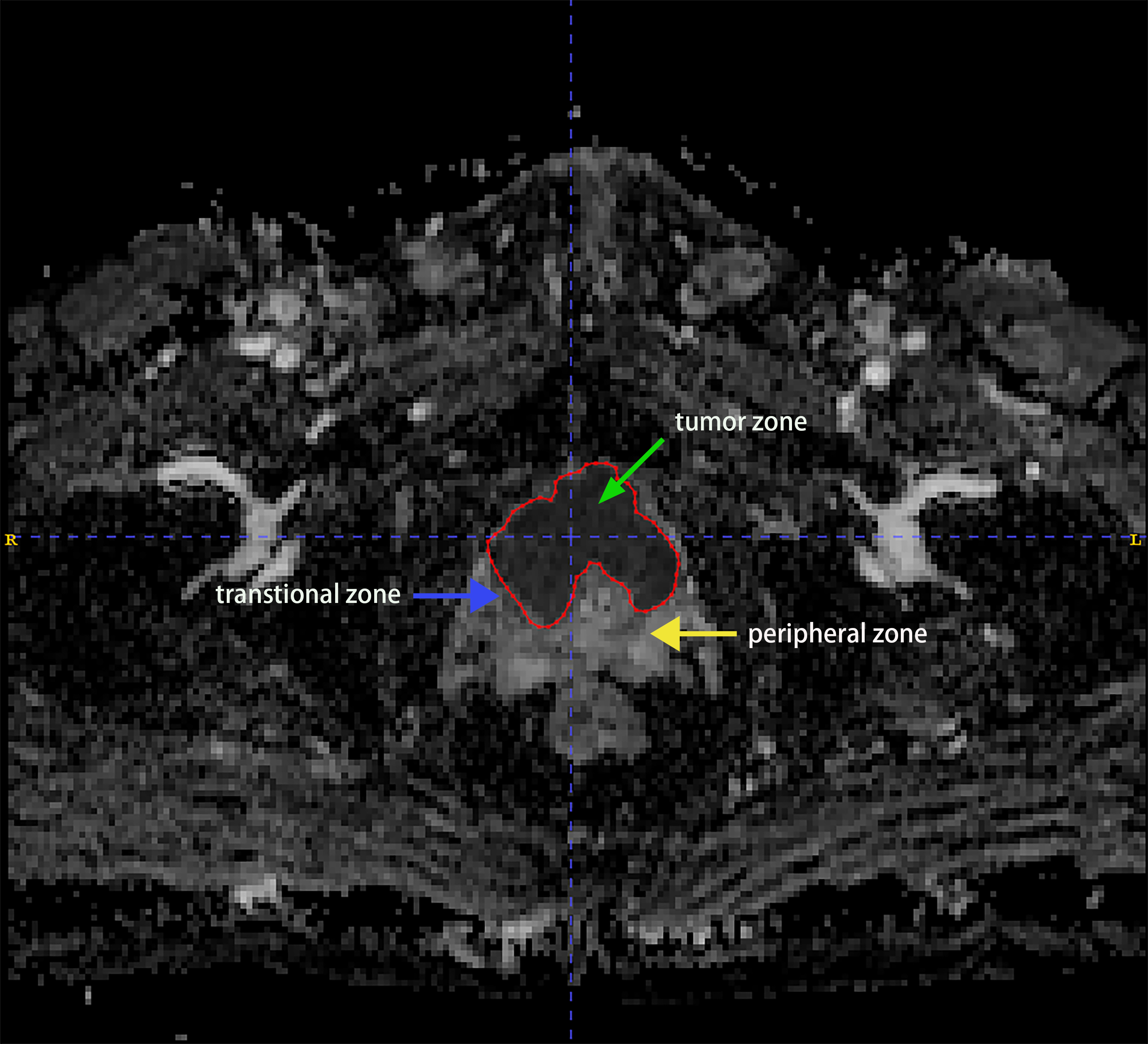

Supplement: Supplementary file 2 — Additional file 2. [file 12672_2023_752_MOESM2_ESM.png]

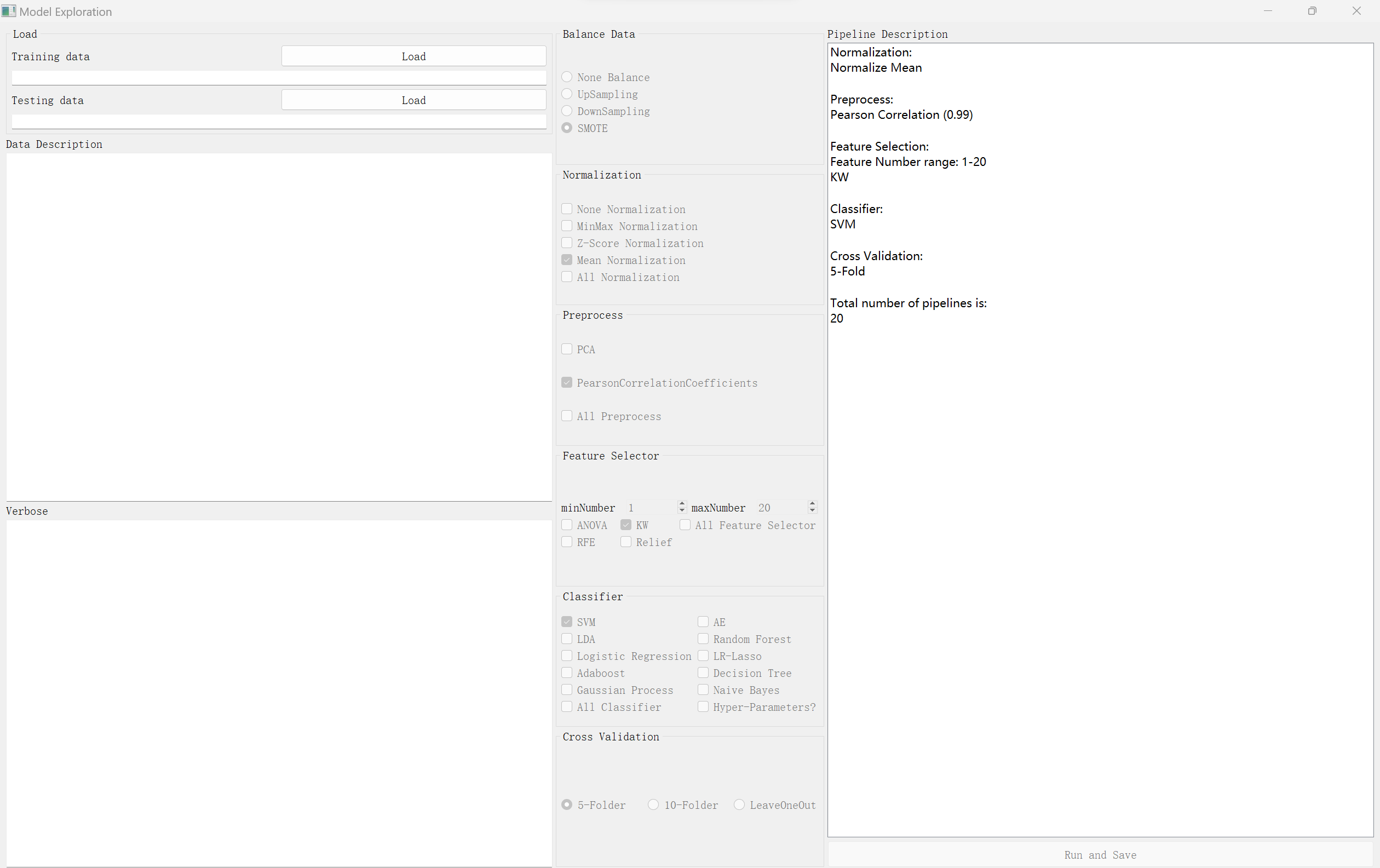

Supplement: Supplementary file 3 — Additional file 3. [file 12672_2023_752_MOESM3_ESM.png]
