# Supplementary material for: Multiparametric MRI radiomics in prostate cancer for predicting Ki-67 expression and Gleason score: a multicenter retrospective study
Source: Discov Oncol. 2023 Jul 20;14:133. doi: 10.1007/s12672-023-00752-w (PMC10361451; doi:10.1007/s12672-023-00752-w)
Supplement: Supplementary file 4 — Additional file 4. [file 12672_2023_752_MOESM4_ESM.docx]

**Table S1. Univariate analysis of prostate cancer death events based on training cohort**

| Variable | β Coefficient | OR(95%CI) | P value |
| --- | --- | --- | --- |
| Age  Gleason Score  Bone metastasis  tPSA  fPSA  BMI  Ki-67 | 1.801  2.768  1.722  0.032  0.106  -1.207  1.906 | 6.06(1.91-19.22)  15.93(5.83-43.49)  5.88(2.48-13.93)  1.03(1.02-1.05)  1.11(1.06-1.17)  0.30(0.13-0.67)  6.73(2.89-15.67) | 0.002  0.001  0.000  0.000  0.000  0.003  0.001 |

CI, confidence interval; OR, odds ratio; BMI, body mass index; The provided bold values mean P-value < 0.05.
