# Supplementary material for: Multiparametric MRI radiomics in prostate cancer for predicting Ki-67 expression and Gleason score: a multicenter retrospective study
Source: Discov Oncol. 2023 Jul 20;14:133. doi: 10.1007/s12672-023-00752-w (PMC10361451; doi:10.1007/s12672-023-00752-w)
Supplement: Supplementary file 5 — Additional file 5. [file 12672_2023_752_MOESM5_ESM.docx]

**Table S2. Multivariate analysis of prostate cancer mortality based on training cohort**

| Variable | β Coefficient | OR(95%CI) | P value |
| --- | --- | --- | --- |
| Age  Gleason Score  Bone metastasis  tPSA  fPSA  BMI  Ki-67 | 2.152  2.278  0.547  0.019  0.036  -0.607  0.974 | 8.60(1.86-39.71)  9.76(2.47-38.61)  1.73(0.41-7.20)  1.02(0.99-1.05)  1.04(0.94-1.15)  0.55(0.17-1.71)  2.65(0.67-10.52) | 0.006  0.001  0.453  0.173  0.484  0.299  0.166 |

CI, confidence interval; OR, odds ratio; BMI, body mass index; The provided bold values mean P-value < 0.05.
